# Hospital-level intracranial pressure monitoring utilization and functional outcome in severe traumatic brain injury: a post hoc analysis of prospective multicenter observational study

**DOI:** 10.1186/s13049-020-00825-7

**Published:** 2021-01-06

**Authors:** Tomoya Okazaki, Kenya Kawakita, Yasuhiro Kuroda

**Affiliations:** Emergency Medical Center, Kagawa University Hospital, 1750-1 Ikenobe, Kita, Miki, Kagawa 761-0793 Japan

**Keywords:** Traumatic brain injury, Hospital-level intracranial pressure monitoring utilization, Patient-level intracranial pressure monitoring utilization, Functional outcome

## Abstract

**Background:**

Several observational studies have shown that hospital-level intracranial pressure (ICP) monitoring utilization varies considerably in patients with severe traumatic brain injury (TBI). However, the relationship between hospital-level ICP monitoring utilization and clinical functional outcomes is unknown. This study examined whether patients with severe TBI treated at hospitals with high ICP monitoring utilization have better functional outcomes.

**Methods:**

A post hoc analysis of the data from a prospective multicenter cohort study in Japan was undertaken, and included severe TBI patients (Glasgow Come Scale score ≤ 8). The primary exposure was hospital-level ICP monitoring utilization. Patients treated at hospitals with more than 80% ICP monitoring utilization were assigned to a high group and the others to a low group. The primary endpoint was a favorable functional outcome at 6 months after injury, defined as a Glasgow Outcome Scale score of good recovery or moderate disability. We conducted multiple logistic regression analyses adjusted for potential confounders.

**Results:**

Of the 427 included patients, 60 were assigned to the high group and 367 to the low group. Multiple logistic regression analysis revealed that patients in the high group had significantly better functional outcome (adjusted odds ratio [OR]: 2.36; 95% confidence interval [CI]: 1.17–4.76; *p* = 0.016). Multiple logistic regression analysis adjusted for additional confounders supported this result (adjusted OR: 2.30; 95% CI: 1.07–4.92; *p* = 0.033).

**Conclusion:**

Treatment at hospitals with high ICP monitoring utilization for severe TBI patients could be associated with better functional outcome.

**Supplementary Information:**

The online version contains supplementary material available at 10.1186/s13049-020-00825-7.

## Background

Traumatic brain injury (TBI) is a leading cause of trauma-related death or disability and confers a serious social burden worldwide [1]. Severe TBI, usually defined on the basis of a Glasgow Coma Scale (GCS) score of less than or equal to 8, is especially associated with high mortality and unfavorable functional outcomes [2].

Elevated intracranial pressure (ICP) commonly occurs during the acute phase of TBI management and is both, a cause and consequence of secondary brain injury that leads to unfavorable functional outcomes [3, 4]. ICP monitoring is significantly associated with reduced short- and long-term mortality [5, 6]; however, there is insufficient evidence that the utilization of patient-level ICP monitoring can contribute to improved functional outcomes [7, 8].

Several observational studies have shown that hospital-level ICP monitoring utilization for severe TBI varies dramatically [5, 9, 10] and ranges from 9.6 to 65.2%, as reported in a study from Los Angeles [9]. Another study from Japan reported utilization in the range of 0 to 100% [10]. However, the relationship between hospital-level ICP monitoring utilization and clinical outcomes is controversial [5, 9].

This study was conducted to examine whether severe TBI patients treated at hospitals with high ICP monitoring utilization have better functional outcomes. The study utilized data from the Japan Neurotrauma Data Bank (JNTDB) Project 2015.

## Materials and methods

### Study design and setting

This study involved a post hoc analysis of data from the JNTDB Project 2015, a prospective multicenter cohort study that was conducted in 32 hospitals in Japan between April 2015 and March 2017. All of the participating institutions had a neurosurgical department and neurosurgeons who were actively involved in the management of TBI patients. The protocol was approved by the Institutional Review Board of each hospital, and all patients or their proxies provided written informed consent. The JNTDB Project 2015 enrolled TBI patients with a GCS score of 8 or lower within 48 h after injury or those requiring craniotomy, regardless of their GCS score. Study participants were followed up until hospital discharge or 6 months after the injury. The details of JNTDB Project 2015 and other JNTDB projects have been described in previous reports [11–13].

### Selection of participants

For the purposes of this study, we included adult patients with severe TBI (GCS score ≤ 8, age ≥ 18 years) without treatment restrictions and an Abbreviated Injury Scale score of 6, and excluded those with missing data for the ICP monitoring utilization, Glasgow Outcome Scale (GOS) score at 6 months after injury, age, GCS score on admission, pupillary reflex, hypotension on admission (systolic blood pressure < 90 mmHg), Marshall Computed Tomography (CT) classification, Injury Severity Score (ISS), and hospital type (university or non-university). Moreover, patients treated at hospitals with fewer than 5 patients were excluded because of the hospital-level analyses designed for this study.

### Data collection

The following data were collected for analysis: the ICP monitoring use, hospital type, age, sex, prescription for anticoagulant drugs, prescription for antiplatelet drugs, GCS on admission, pupillary reflex (none, one, or both), hypotension on admission, body temperature on admission, Marshall CT classification, Abbreviated Injury Scale score, ISS, isolated TBI (defined as any Abbreviated Injury Scale score of 0, other than that for the head), cause of injury, and 6-month post-injury GOS score.

### Exposure

The primary exposure was the hospital-level ICP monitoring utilization, which was calculated as the ratio of the patients treated with ICP monitoring to the total number of patients who met the criteria for this study at each hospital. We classified hospitals into two categories based on their ICP monitoring utilization: patients treated at hospitals with more than and less than 80% ICP monitoring utilization were assigned to a “high” and a “low” group, respectively. The 80% cutoff was based on previous studies in the United States which found that, between 2007 and 2013, nearly 80% of severe TBI patients were treated with ICP monitoring [14, 15].

The secondary exposure was the patient-level ICP monitoring utilization. Patients treated with and without ICP monitoring were assigned to an “ICP (+)“and an “ICP (−)” group, respectively.

### Outcome measures

The primary endpoint was a favorable functional outcome at 6 months after injury, defined as a GOS score of 4 or 5 (moderate disability or good recovery) [16]. The other three categories based on the GOS score were: 1, death; 2, persistent vegetative state; and 3, severe disability.

### Statistical analysis

Continuous variables were analyzed using the Mann–Whitney *U* test, and categorical comparisons were conducted with the Fisher’s exact or chi-square test.

To clarify selection biases, we initially compared the baseline characteristics of patients included into the final analysis and those who were excluded due to missing data or fewer cases. Thereafter, we calculated the hospital-level ICP monitoring utilization, and compared the baseline characteristics and functional outcome at 6 months after injury in the high and low groups. We conducted a multiple logistic regression analysis that was adjusted for age, GCS motor score, and pupillary reflex to examine the association of hospital-level ICP monitoring utilization with the primary outcome (model 1). The abovementioned factors were selected on the basis of the International Mission for Prognosis and Analysis of Clinical Trials in TBI (IMPACT) Core model [17–19]. We performed another multiple logistic regression analysis adjusted for the age, GCS motor score, pupillary reflex, hypotension on admission, Marshall CT classification, and ISS, as well as the hospital type as a hospital-level variable (model 2). These factors were determined by referring to the IMPACT Extended model [17–19] and clinical plausible.

Furthermore, to evaluate the validity of the threshold between the high and the low group, we conducted sensitivity analysis based on further subclassification of the hospital-level ICP monitoring utilization. We ranked hospitals into five categories based on their ICP monitoring utilization rates (0–19%, 20–39%, 40–59%, 60–79%, and 80–100%) and conducted multiple logistic regression analyses adjusted for the variables of the model 1 and model 2.

To examine a possible association between the secondary exposure and the primary outcome, we compared the baseline characteristics and functional outcome at 6 months after injury between the ICP (+) and the ICP (−) group. After adjusting for the variables of the model 1 and model 2, we conducted multiple logistic regression analyses.

A two-sided *P-*value less than 0.05 was considered statistically significant. Missing data were not replaced or estimated. All statistical analyses were conducted in EZR (Saitama Medical Center, Jichi Medical University, Saitama, Japan) [20].

## Results

**Fig. 1:**
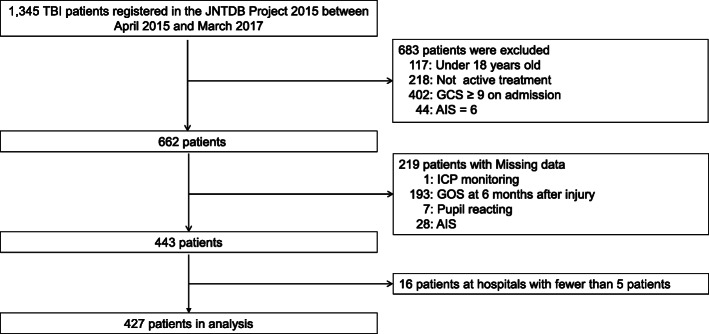
Patient flow diagram. TBI traumatic brain injury, JNTDB Japan Neurotrauma Data Bank, GCS Glasgow Coma Scale, AIS Abbreviated Injury Scale, ICP intracranial pressure, GOS Glasgow Outcome scale

**Fig. 2:**
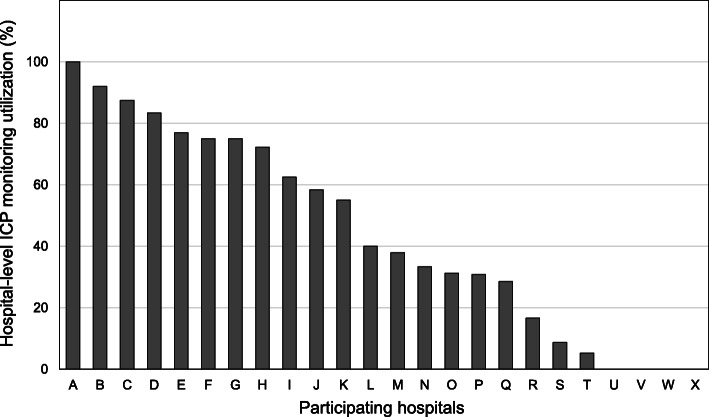
Inter-hospital variation of intracranial pressure monitoring utilization

The JNTDB Project 2015 enrolled 1345 patients during the study period. Of the 662 potential participants, 427 (65%) met the eligibility criteria for this study (Fig. 1). A comparison of the baseline characteristics between the patients included in the final analysis and those who were excluded are shown in Additional Table 1. The ICP monitoring utilization at each hospital remarkably ranged from 0 to 100% (Fig. 2).

Sixty patients (14%) who were treated at four hospitals with ICP monitoring utilization of more than 80% were assigned to the high group, and the remaining 367 patients were assigned to the low group.

Table 1 shows the clinical characteristics and outcomes of the study participants. The median age in this study population was 65 years (interquartile [IQR] 46–77), and 282 (66%) participants were male. Six months after the TBI, 137 (32%) patients had a favorable functional outcome. There were significant differences between the high and the low groups with regard to the distribution of the Marshall CT classification and the ISS. Moreover, compared with the low group, the high group was more frequently treated with ICP monitoring and at the university hospitals, and had a high proportion of favorable functional outcome.

**Table 1:** Baseline characteristics and outcomes, Treatment at the hospitals with high ICP monitoring utilization vs. low utilization

Variables	Overall *n* = 427	High group *n* = 60	Low group *n* = 367	*P* value	Missing data
Age, years, median [IQR]	65 [46, 77]	65 [43, 78]	66 [47, 77]	0.755	0
Male sex, n (%)	282 (66)	36 (60)	246 (67)	0.305	0
Prescribing anticoagulant drugs, n (%)	19 (4.4)	2 (3.3)	17 (4.6)	1	0
Prescribing antiplatelet drugs, n (%)	36 (8.4)	7 (11.7)	29 (7.9)	0.319	0
Glasgow Coma Scale score, median [IQR]					0
Overall score	6 [3, 7]	6 [3, 7]	6 [3, 7]	0.305	
Motor score	3 [1, 4]	4 [1, 5]	3 [1, 4]	0.288	
Pupillary reflex, n (%)				0.334	0
None	110 (26)	13 (22)	97 (26)		
One	91 (21)	17 (28)	74 (20)		
Both	226 (53)	30 (50)	196 (53)		
Hypotension, n (%)	60 (14)	7 (12)	53 (14)	0.69	0
Body temperature, °C, median [IQR]	36.2 [35.8, 36.8]	36.2 [35.9, 36.9]	36.2 [35.8, 36.8]	0.367	34
Marshall CT classification, n (%)				0.002	0
diffuse injury I	9 (2.1)	1 (1.7)	8 (2.2)		
diffuse injury II	96 (22.5)	12 (20.0)	84 (22.9)		
diffuse injury III	48 (11.2)	3 (5.0)	45 (12.3)		
diffuse injury IV	11 (2.6)	2 (3.3)	9 (2.5)		
evacuated mass	175 (41.0)	38 (63.3)	137 (37.3)		
non-evacuated mass	88 (20.6)	4 (6.7)	84 (22.9)		
Injury Severity Score, median [IQR]	25 [25, 35]	25 [18, 29]	25 [18, 29]	0.045	0
Isolated traumatic brain injury, n (%)	173 (41)	28 (47)	145 (40)	0.322	0
Cause of injury, n (%)				0.344	2
Motor vehicle	27 (6.3)	26 (7.1)	1 (1.7)		
Motorcycle	53 (12.4)	45 (12.3)	8 (13.6)		
Bicycle	34 (8.0)	28 (7.7)	6 (10.2)		
Pedestrian	90 (21.1)	82 (22.4)	8 (13.6)		
High-level fall	115 (27.0)	95 (26.0)	20 (33.9)		
Ground-level fall	79 (18.5)	68 (18.6)	11 (18.6)		
Others	18 (4.2)	22 (6.0)	5 (8.5)		
Treatment with ICP monitoring, n (%)	192 (45)	55 (92)	137 (37)	< 0.001	0
Treatment at university hospitals, n (%)	257 (60)	45 (75)	212 (58)	0.015	0
GOS at 6 months after injury, n (%)				0.074	0
Good recovery	75 (17.6)	12 (20.0)	63 (17.2)		
Moderate disability	62 (14.5)	15 (25.0)	47 (12.8)		
Severe disability	40 (9.4)	7 (11.7)	33 (9.0)		
Vegetable state	41 (9.6)	4 (6.7)	37 (10.1)		
Death	209 (48.9)	22 (36.7)	187 (51.0)		
Favorable outcome at 6 months after injury, n (%)	137 (32)	27 (45)	110 (30)	0.025	0

*IQR* interquartile range, *CT* computed tomography, *ICP* intracranial pressure, *GOS* Glasgow Outcome Scale

The results of the multiple logistic regression analyses are summarized in Table 2. The model 1 revealed an association between the high group and increased favorable functional outcome (adjusted odds ratio [OR]: 2.36; 95% confidence interval (CI): 1.17–4.76; *p* = 0.016). Moreover, this association was observed in the model 2 that was adjusted for the age, GCS motor score, pupillary reflex, hypotension on admission, Marshall CT classification, ISS, and hospital type (adjusted OR: 2.30; 95% CI: 1.07–4.92; *p* = 0.033).

**Table 2:** Association between hospital-level ICP monitoring utilization and favorable functional outcome

Variables	Model 1		Model 2	
	Adjusted OR (95% CI)	*P* value	Adjusted OR (95% CI)	*P* value
Higher hospital-level ICP monitoring utilization	2.36 (1.17–4.76)	0.016	2.30 (1.07–4.92)	0.033
Age, per year	0.94 (0.93–0.96)	< 0.001	0.93 (0.92–0.95)	< 0.001
Glasgow Coma Scale Motor score, per point	1.50 (1.25–1.80)	< 0.001	1.40 (1.16–1.69)	< 0.001
Pupillary reflex				
None	Reference	–	Reference	–
One	5.27 (2.11–13.20)	< 0.001	4.53 (1.72–11.90)	0.002
Both	10.00 (4.19–24.00)	< 0.001	7.28 (2.86–18.50)	< 0.001
Hypotension on admission	–	–	0.29 (0.09–0.96)	0.042
Marshall CT classification	–	–		
Diffuse injury I	–	–	Reference	–
Diffuse injury II	–	–	0.16 (0.00–5.12)	0.300
Diffuse injury III	–	–	0.05 (0.00–1.80)	0.100
Diffuse injury IV	–	–	0.10 (0.00–4.37)	0.230
Evacuated mass	–	–	0.10 (0.00–3.08)	0.190
Non-evacuated mass	–	–	0.12 (0.00–3.76)	0.220
Injury Severity Score, per point	–	–	0.95 (0.93–0.98)	< 0.001
Treatment at university hospitals	–	–	0.97 (0.55–1.69)	0.902

*OR* odds ratio, *CI* confidence interval, *ICP* intracranial pressure, *CT* computed tomography

**Fig. 3:**
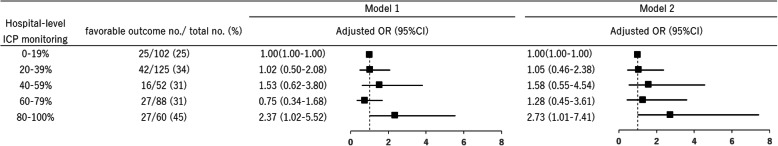
Association between the subclassification of hospital-level ICP monitoring utilization and favorable functional outcome. ICP intracranial pressure, OR odds ratio, CI confidence interval. Model 1 was adjusted for age, Glasgow Coma Scale Motor score, and pupillary reflex. Model 2 was adjusted for age, Glasgow Coma Scale Motor score, pupillary reflex, hypotension an admission, Marshall CT classification, Injury Severity Score, and hospital type

The intergroup analysis based on the subclassification by hospital-level ICP monitoring utilization showed that, with regard to the 0–19% group, only the 80–100% group was significantly associated with increased favorable functional outcome – both in the model 1 and model 2 multiple logistic regression analyses (Fig. 3).

The results of patient-level intergroup comparison of the ICP (+) and ICP (−) groups are shown in Table 3, and showed significant differences in the pupillary reflex, hypotension, the distribution of the Marshall CT classification, hospital type, and the distribution of GOS at 6 months after injury. After adjusting the covariates, neither the model 1 nor model 2 multiple logistic regression analyses showed an association between patient-level ICP monitoring utilization and better functional outcome (Table 4).

**Table 3:** Baseline characteristics and outcomes, treatment with vs. without ICP monitoring

Variables	Overall *n* = 427	ICP (+) group *n* = 192	ICP (−) group *n* = 235	*P* value	Missing data
Age, years, median [IQR]	65 [46, 77]	65 [45, 76]	66 [50, 79]	0.173	0
Male sex, n (%)	282 (66)	121 (63)	161 (69)	0.259	0
Prescribing anticoagulant drugs, n (%)	19 (4.4)	7 (3.6)	12 (5.1)	0.492	0
Prescribing antiplatelet dugs, n (%)	36 (8.4)	17 (8.9)	19 (8.1)	0.861	0
Glasgow Coma Scale score, median [IQR]					0
Overall score	6 [3, 7]	6 [3, 7]	6 [3, 7]	0.566	
Motor score	3 [1, 4]	3 [1, 4]	4 [1, 4.]	0.614	
Pupillary reflex, n (%)				0.002	0
None	110 (26)	39 (20)	71 (30)		
One	91 (21)	55 (29)	36 (15)		
Both	226 (53)	98 (51)	128 (55)		
Hypotension, n (%)	60 (14)	16 (8)	44 (19)	0.002	0
Body temperature, °C, median [IQR]	36.2 [35.8, 36.8]	36.2 [35.8, 36.8]	36.2 [35.8, 36.8]	0.876	34
Marshall CT classification, n (%)				0.001	0
diffuse injury I	9 (2.1)	2 (1.0)	7 (3.0)		
diffuse injury II	96 (22.5)	29 (15.1)	67 (28.5)		
diffuse injury III	48 (11.2)	24 (12.5)	24 (10.2)		
diffuse injury IV	11 (2.6)	5 (2.6)	6 (2.6)		
evacuated mass	175 (41.0)	98 (51.0)	77 (32.8)		
non-evacuated mass	88 (20.6)	34 (17.7)	54 (23.0)		
Injury Severity Score, median [IQR]	25 [25, 35]	26 [25, 34]	25 [22, 37]	0.441	0
Isolated traumatic brain injury, n (%)	173 (41)	77 (40)	96 (41)	0.921	0
Cause of injury, n (%)				0.703	2
Motor vehicle	27 (6.3)	12 (6.3)	15 (6.4)		
Motorcycle	53 (12.4)	25 (13.1)	28 (12.0)		
Bicycle	34 (8.0)	42 (22.0)	48 (20.5)		
Pedestrian	90 (21.1)	17 (8.9)	17 (7.3)		
High-level fall	115 (27.0)	54 (28.3)	61 (26.1)		
Ground-level fall	79 (18.5)	28 (14.7)	51 (21.8)		
Others	18 (4.2)	13 (6.8)	14 (6.0)		
Treatment at university hospitals, n (%)	257 (60)	146 (76.0)	111 (47.2)	< 0.001	0
GOS at 6 months after injury, n (%)				0.001	0
Good recovery	75 (17.6)	28 (14.6)	47 (20.0)		
Moderate disability	62 (14.5)	36 8 (18.8)	26 (11.1)		
Severe disability	40 (9.4)	21 (10.9)	19 (8.1)		
Vegetable state	41 (9.6)	27 (14.1)	14 (6.0)		
Death	209 (48.9)	80 (41.7)	129 (54.9)		
Favorable outcome at 6 months after injury, n (%)	137 (32)	64 (33)	73 (31)	0.677	0

*IQR* interquartile range, *CT* computed tomography, *ICP* intracranial pressure, *GOS* Glasgow Outcome Scale

**Table 4:** Association between patient-level ICP monitoring utilization and favorable functional outcome

Variables	Model 1		Model 2	
	Adjusted OR (95% CI)	*P* value	Adjusted OR (95% CI)	*P* value
Treatment with ICP monitoring	0.96 (0.57–1.59)	0.863	1.08 (0.60–1.94)	0.791
Age, per year	0.94 (0.93–0.96)	< 0.001	0.94 (0.92–0.95)	< 0.001
Glasgow Coma Scale Motor score, per point	1.52 (1.27–1.82)	< 0.001	1.41 (1.16–1.70)	< 0.001
Pupillary reflex				
None	Reference	–	Reference	–
One	5.40 (2.15–13.60)	< 0.001	4.74 (1.79–12.50)	0.002
Both	9.59 (4.05–22.70)	< 0.001	7.26 (2.89–18.30)	< 0.001
Hypotension on admission	–	–	0.31 (0.09–1.03)	0.056
Marshall CT classification	–	–		
Diffuse injury I	–	–	Reference	–
Diffuse injury II	–	–	0.16 (0.01–4.81)	0.294
Diffuse injury III	–	–	0.05 (0.00–1.65)	0.094
Diffuse injury IV	–	–	0.11 (0.00–4.44)	0.239
Evacuated mass	–	–	0.11 (0.00–3.09)	0.192
Non-evacuated mass	–	–	0.11 (0.00–3.29)	0.203
Injury Severity Score, per point	–	–	0.95 (0.93–0.98)	< 0.001
Treatment at university hospitals	–	–	1.01 (0.57–1.79)	0.975

*OR* odds ratio, *CI* confidence interval, *ICP* intracranial pressure, *CT* computed tomography

## Discussion

To our knowledge, this is the first study to assess the association of hospital-level ICP monitoring utilization with functional outcome in patients with severe TBI. In this study, we found that treatment at hospitals with ICP monitoring utilization of more than 80% was significantly associated with better functional outcome at 6 months after injury in patients with severe TBI. However, the patient-level ICP monitoring utilization was unrelated to functional outcome.

Two previous studies assessed the association between hospital-level ICP monitoring utilization and in-hospital mortality [5, 9]. Alali et al. showed that patients with severe TBI at hospitals with ICP monitoring utilization rates of more than 16.1% had significantly improved outcomes than those at hospitals with rates of less than 8.3% [5]. This threshold was considerably lower than our threshold of 80%, and the discrepancy may be attributable to the difference in the outcomes. Whereas we set the 6-month functional outcome as the primary outcome, Alali et al. used an entirely different metric – in-hospital mortality. In general, it is more difficult to improve functional outcome than to decrease mortality, as several previous studies on neurocritical care have suggested [21, 22]; thus, it may be reasonable that the clinical threshold of better functional outcome is higher than that of decreased mortality. A study by Dawes and colleagues did not find an association of high hospital-level ICP monitoring utilization with improved outcome [9]. We excluded patients with any Abbreviated Injury Scale score of 6, who were not expected to survive; however, Dawes et al. did not exclude their patients based on this criteria and, therefore, the patients in their study were considered to be too severely ill to obtain benefits from hospitals with high hospital-level ICP monitoring utilization.

We did not evaluate the factors at the hospitals with high ICP monitoring utilization to assess which worked well on the functional outcome. The patient-level ICP monitoring utilization may explain this association, because the high group was more frequently treated with ICP monitoring than the low group (92% vs 37%, *p* <  0.001). However, our patient-level analysis and previous studies did not support this suspicion. Furthermore, ICP monitoring is not a therapeutic intervention but is merely a monitoring system. Therefore, whether patients can obtain net benefits from ICP monitoring apparently depends on whether their physicians can effectively utilize ICP information in a timely manner. Physicians at hospitals with high ICP monitoring utilization may have better-quality opportunities to develop their skills in optimizing ICP monitoring. Perhaps, it is for this reason that patients treated at hospitals with high ICP monitoring utilization had better functional outcomes. Further investigation is needed to validate the preliminary findings of this study and to validate the role of ICP monitoring utilization in the severe TBI patient population.

The present study has several limitations. First, this was a post hoc analysis of a prospective multicenter cohort study. We conducted multiple logistic regression analyses that were adjusted for clinically important factors; however, selection bias and uncontrolled confounding variables may have influenced the results due to the observational study design. Second, we excluded 219 of the 662 potentially eligible patients due to missing data for important variables (Fig. 1). Thus, the baseline characteristics of the patients included in the final analysis were different from those of the excluded patients (Table E1, web-only appendices)), which may have led to some selection biases. Third, there were no specific management protocols for severe TBI in the JNTDB study, and treatment strategies depended crucially on each institution; thus, this may have led to additional biases. Furthermore, we did not consider hospital-level factors other than the utilization rate of ICP monitoring and hospital type (university or non-university). Finally, there are two possible generalization problems: (1) as these results were derived from the JNTDB Project 2015 that was conducted in the Japanese population, the findings may not be generalizable to other countries and (2) the reason for the distribution trend of ICP monitoring utilization across the study centers was unclear, and therefore, it is unknown whether the generalization of the results to other settings would be feasible.

## Conclusions

In summary, treatment at hospitals with high ICP monitoring utilization for severely ill patients with TBI was associated with better functional outcome.

## Supplementary Information

**Additional file 1: Additional Table 1.** Baseline characteristics, inclusion vs. exclusion group

## Data Availability

The datasets used in the current study are available from the corresponding author on reasonable request.

## Abbreviations

TBI: Traumatic brain injury
GCS: Glasgow Coma Scale
ICP: Intracranial pressure
JNTDB: Japan Neurotrauma Data Bank
GOS: Glasgow Outcome Scale
CT: Computed Tomography
ISS: Injury Severity Score
IMPACT: International Mission for Prognosis and Analysis of Clinical Trials
