# Supplementary material for: Hospital-level intracranial pressure monitoring utilization and functional outcome in severe traumatic brain injury: a post hoc analysis of prospective multicenter observational study
Source: Scand J Trauma Resusc Emerg Med. 2021 Jan 6;29:5. doi: 10.1186/s13049-020-00825-7 (PMC7789401; doi:10.1186/s13049-020-00825-7)
Supplement: Supplementary file 1 — Additional file 1: Additional Table 1. Baseline characteristics, inclusion vs. exclusion group [file 13049_2020_825_MOESM1_ESM.docx]

**Additional Table1.** Baseline characteristics, inclusion vs. exclusion group

| Variables | | Inclusion  n=427 | Exclusion  n=235 | P value | Missing data |
| --- | --- | --- | --- | --- | --- |
| Age, years, median [IQR] | | 65 [46, 77] | 72 [55, 79] | 0.002 | 0 |
| Male sex, n (%) | | 282 (66) | 171 (73) | 0.081 | 0 |
| Prescribing anticoagulant drugs, n (%) | | 19 (4.4) | 26 (11.1) | 0.002 | 0 |
| Prescribing antiplatelet dugs, n (%) | | 36 (8.4) | 32 (13.6) | 0.044 | 0 |
| Glasgow Coma Scale score, median [IQR] | |  |  |  | 0 |
|  | Overall score | 6 [3, 7] | 6 [3, 7] | 0.645 |  |
|  | Motor score | 3 [1, 4] | 3 [1, 4] | 0.904 |  |
| Pupil reacting, n (%) | |  |  | 0.094 | 7 |
|  | None | 110 (26) | 42 (18) |  |  |
|  | One | 91 (21) | 50 (22) |  |  |
|  | Both | 226 (53) | 136 (60) |  |  |
| Hypotension, n (%) | | 60 (14) | 15 (6) | 0.003 | 0 |
| Body temperature, ℃, median [IQR] | | 36.2 [35.8, 36.8] | 36.3 [35.8, 36.9] | 0.145 | 9 |
| Marshall CT classification, n (%) | |  |  | 0.028 | 0 |
|  | diffuse injury I | 9 (2) | 6 (3) |  |  |
|  | diffuse injury II | 96 (23) | 60 (26) |  |  |
|  | diffuse injury III | 48 (11) | 8 (3) |  |  |
|  | diffuse injury IV | 11 (3) | 7 (3) |  |  |
|  | evacuated mass | 175 (41) | 98 (42) |  |  |
|  | non-evacuated mass | 88 (21) | 56 (24) |  |  |
| Injury Severity Score, median [IQR] | | 25 [25, 35] | 25 [16, 29] | <0.001 | 28 |
| Isolated traumatic brain injury, n (%) | | 173 (41) | 108 (46) | 0.189 | 0 |
| Cause of injury, n (%) | |  |  | 0.086 | 2 |
|  | Motor vehicle | 27 (6.3) | 13 (5.6) |  |  |
|  | Motorcycle | 53 (12.4) | 18 (7.7) |  |  |
|  | Bicycle | 34 (8.0) | 22 (9.4) |  |  |
|  | Pedestrian | 90 (21.1) | 42 (17.9) |  |  |
|  | High-level fall | 115 (27.0) | 56 (23.9) |  |  |
|  | Ground-level fall | 79 (18.5) | 65 (27.8) |  |  |
|  | Others | 27 (6.4) | 17 (7.3) |  |  |
| ICP monitoring, n (%) | | 192 (45) | 106 (45) | 0.935 | 1 |
| Treatment at university hospitals, n (%) | | 257 (60) | 144 (61) | 0.804 | 0 |

IQR interquartile range, CT computed tomography, ICP intracranial pressure
